# Public Perceptions of Climate Change and Health—A Cross-Sectional Survey Study

**DOI:** 10.3390/ijerph20021464

**Published:** 2023-01-13

**Authors:** Katharina van Baal, Stephanie Stiel, Peter Schulte

**Affiliations:** 1 Institute for General Practice and Palliative Care, Hannover Medical School, Carl-Neuberg-Straße 1, 30625 Hannover, Germany; 2 Länger Besser Leben. Institute, University of Applied Sciences Weserbergland, Am Stockhof 2, 31785 Hameln, Germany

**Keywords:** climate change, planetary health, public perception, public health

## Abstract

Climate change is inseparably linked to human health. Although there is growing awareness of the threats to human health caused by climate change, it remains unclear how the German population perceives the relevance of climate change and its health consequences. Between May and September 2022, German residents were invited to participate in a cross-sectional online survey that explored three content areas: (1) the relevance of climate change, (2) health risks in connection with climate change and (3) collective and individual options for action against climate change. A total of 697 full data sets were collected for analysis (72% female, 51% ≥55 years old). The majority of participants agreed that human-induced climate change exists (85%), and that it has an impact on human health (83%). They also perceived the global population to be more strongly impacted by climate change than themselves (89% versus 68%). Most participants (76%) claimed to personally contribute to climate protection and 23% felt that their city or council contributed to climate protection. Although the majority of participants saw climate change as a threat to human health, they perceived other population groups to be most strongly affected. Cognitive dissonance might explain this lack of individual concern and one approach to addressing such distorted perceptions might be the dissemination of appropriate risk communication with health professionals involved in the communication.

## 1. Introduction

Currently, climate change is the greatest hazard to humankind, threatening our natural sources of life and the survival of our civilisation [1,2,3]. Although countries have committed to limiting global warming to well below 2 °C (as part of the Paris Agreement), global greenhouse gas emissions continue to increase [1]. In fact, planetary boundaries, defined as thresholds that should not be exceeded in order to ensure sustainable living conditions, have already been crossed [4]. The global scientific community agrees that global warming must be contained within 1.5 °C to maintain decent living conditions. Recently, a haunting warning was released, specifying the growing magnitude of climate-related disasters resulting in global human suffering [5]. Despite this, the climate is changing faster than anticipated and the 1.5 °C threshold is predicted to be surpassed by 2040 or even earlier, with potentially catastrophic effects [3,5,6]. Vulnerable populations such as the elderly and young children will be most affected, resulting in growing inequalities [7].

Climate change contributes significantly to human morbidity and mortality and has direct effects on individuals and on global and public health [1,6,8,9]. Increasing temperatures and heat periods, extreme weather events, air pollution, water and food insecurity and changes in the spread patterns of vector-associated diseases are effects of climate change with a direct or indirect impact on human health. Science has confirmed that these changes affect health in complex ways, resulting in, among other things, heat injuries, infectious diseases, allergies, malnutrition, and mental illnesses [6,9,10].

In the international medical community, there is a growing awareness of the threats to human health caused by climate change [11,12,13,14,15]. As a result, ideas for the sustainable and efficient management of resources in health care settings have been proposed [14], the carbon footprint of primary care practices has been measured [15] and the attitude of medical doctors towards climate protection measures in outpatient practices has been assessed [13]. In addition, the population in Germany [16] and in other countries [7,17] has recently shown increasing concern about climate change and its implications on health. However, few people understand the types of harm climate change cause on health and know precisely who is most likely to be affected [7]. Despite this growing concern about climate change itself and its implications on health, few studies have yet assessed public perceptions. Active involvement of the population is crucial for the empowerment of people participating in (health) politics, policy-making, and research [18]. However, in Germany, current public perceptions of the relevance of climate change and the connection between climate change and health remain unclear.

Therefore, the present study aimed at investigating public perceptions of the relevance of climate change, the connection between climate change and health, and options for action against climate change. The specific research questions were:

- How does the public perceive the overall relevance of climate change?
- How does the public perceive the risks and health consequences associated with climate change?
- What are individual and collective options for action against climate change?

## 2. Materials and Methods

### 2.1. Study Design

The present study was a cross-sectional survey study. This research approach promises to reach a large population sample with few distortions and barriers for participation. The design was based on a previous survey by Berger et al. [16].

### 2.2. Study Population

The target group was an open population in a national German sample. There were no inclusion or exclusion criteria. Participants were recruited via the websites, mailing lists and social media accounts of the involved institutions (i.e., health insurance fund BKK24, institutes at Hannover Medical School and the University of Applied Sciences Weserbergland).

### 2.3. Survey

The survey content was based on a questionnaire by Berger et al. [16], which was adapted and supplemented on the basis of the literature and the research questions. The survey was administered as an online survey via the website of the German health insurance fund BKK24. There was an internal pre-test with the staff of the involved institutions. The survey was open for a duration of 20 weeks, from 2 May 2022 to 18 September 2022, and consisted of four major parts:

- five items on demographic variables;
- four items on overall perceptions of climate change;
- seven items on suspected or perceived risks and health consequences associated with climate change;
- eight items on individual and collective options for action against climate change.

The survey contained nominal single-choice questions (e.g., Yes/No), as well as verbal Likert scales ranging from 1 (not at all) to 5 (fully), to determine the extent to which participants agreed with or were concerned about certain aspects of climate change. Free text options were provided for some items to invite elaboration on individual attitudes and perceptions.

### 2.4. Ethical Approval and Consent to Participate

Ethical approval (No. 04/2022-8) was granted by the ethics committee of University of Applied Sciences Weserbergland on 4 April 2022. All participants provided informed consent prior to participating in any study procedure. All data were pseudonymised.

### 2.5. Data Analysis

Data were analysed using version 26 of the Statistical Package for Social Sciences software (SPSS Inc., Chicago, IL, USA). Descriptive analyses included the calculation of means, standard deviations (SDs), medians and interquartile ranges (IQRs). Smaller sample sizes were stated for all items, indicating missing values. Single free text comments on certain survey items were used to describe the quantitative data. Free text comments were categorised according to conventional content analysis by Hsieh and Shannon [19].

## 3. Results

During the survey period (May to September 2022), 1167 people accessed the questionnaire. Of the resulting 1167 data sets, 363 were empty and 107 only included demographic data. These 470 data sets were excluded prior to the data analysis. In total, data from 697 participants were included in the analysis.

The study population included 503 (72.2%) female participants. Table 1 presents further demographic details.

**Figure 1:**
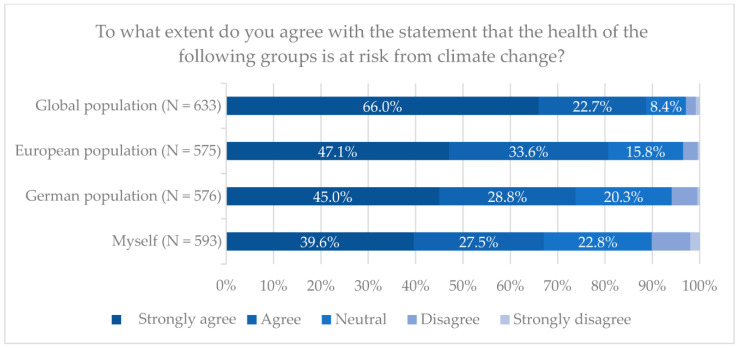
Health at risk from climate change, by population group.

The majority (85.4%) of participants agreed or thought it likely that human-induced climate change exists. A total of 504 (75.7%) participants claimed to be very or somewhat concerned about climate change. Additionally, 82.8% agreed or thought it likely that climate change has an impact on human health. Most (69.9%) participants were strongly or rather concerned about the health consequences of climate change (Table 2). Participants also perceived the global population (88.8% agreed fully or deemed it likely) to be more strongly impacted by climate change than the European population (80.7%), the German population (73.8%) and themselves (67.1%) (Figure 1).

Participants believed that extreme weather events (87.8%), problems with the quality and supply of drinking water (74.2%), and rising sea levels (66.8%) were the three most relevant factors associated with climate change and its health risks. An additional 54 factors were proposed as free text answers. Predominantly, these fell into the categories of hunger/food security (n = 13), flight/migration (n = 10) and war (n = 8). Furthermore, participants ranked accidents and deaths from extreme weather events (61.6%), infectious diseases (56.2%) and skin cancer (51.7) as the top three health risks connected to climate change. A significant proportion (n = 114; 18.2%) had experienced changes in their own health due to climate change (Table 3).

The majority (81.1%) of participants believed that they could personally contribute to climate protection, while 15.3% were unsure and 3.6% thought that they could not contribute (N = 634). Most (77.3%) participants claimed to already be contributing in some way to climate protection, while 17.8% were unsure of their personal contribution and 4.9% claimed that they were not actively contributing (N = 616). Asked to describe their options for personally contributing to climate protection, participants most frequently mentioned actions related to mobility (n = 291), resource management (n = 195) and food procurement (n = 179) (Table 4). The same categories were mentioned most frequently when participants were asked to describe the contributions they were already making to climate protection (Table 4).

The majority (75.1%) of participants believed that their city or council could contribute to climate protection, while 20.7% were unsure and 4.2% did not believe in the possible contribution of their city or council (N = 627). Moreover, 23.3% perceived that their city or council was already taking action for climate protection, whereas 68.4% were unsure about this and 8.3% did not see any action being taken. When participants were asked to describe any concrete contributions to climate protection that their city or council could make, they most frequently mentioned actions related to traffic and mobility (n = 242), modernisation and extension of renewable energy sources (n = 141), and green spaces and planting (n = 139) (Table 5). While fewer actual contributions of the city or council were mentioned, those that were tended to fall into the same categories as the possible contributions (Table 5).

## 4. Discussion

The present study was aimed at investigating public perceptions of the relevance of climate change, the connection between climate change and health, and options for action against climate change. The underlying research questions related to (1) how the public perceives the overall relevance of climate change; (2) how risks and health consequences associated with climate change are perceived, and (3) what individual and collective options for action against climate change exist. The main results regarding these research questions were that the majority of a public population (1) acknowledged the existence of climate change and its implications for human health and were concerned about the associated risks of climate change, (2) perceived that other population groups would be more strongly impacted by climate change than the German population and themselves, and (3) claimed to contribute to climate protection, while noting potential improvements in the climate change mitigating activities of cities and councils.

In the scientific community, there is a consensus that climate change is human-induced and associated with wide-ranging environmental changes that may negatively impact human health. Accordingly, climate change is considered the greatest existential challenge faced by humanity [1,2,8,20]. Despite this, research from 2018 [16] found that the public perception of climate change in Germany was characterised by psychological distance, with most participants downplaying its associated health risks. The authors called for targeted communication measures with comprehensive information and action plans for broad parts of the population, in order to influence risk perception and willingness to act. However, the results of the present study do not show a fundamental alteration in public perception. Concern about climate change and its implications remained high, particularly in relation to extreme weather events. Also, the health impacts of climate change were perceived as stronger for other population groups than for the participants themselves. Possible explanations for this have relevance for both climate change and its implications for health. Climate change and greenhouse gas emissions are not visible, and they are rarely immediately connected with their health risks [21,22]. Even though a scientific consensus exists, concern and sense of urgency vary strongly, often due to a limited understanding of the underlying causes and stakes [21]. In addition, the lack of immediacy is often caused by a geographic and temporal distance between cause and effect of health-related climate change effects [21]. The high complexity of climate change, in terms of its processes, consequences and public controversies (fuelled by politicians and public figures), leads to public questioning of the substantially confirmed scientific findings on climate change and its implications [23,24,25]. Furthermore, cognitive dissonance theory might explain participants’ belief that other groups are and will be more strongly impacted by climate change than themselves. Specifically, dissonance between knowledge, conflicting values, and actual behaviour may contribute to a distortion of understanding, information processing and decision-making [26]. Cognitive dissonance theory has been recognised as influential in clinical medical practice and medical education [27], as well as in the psychology of eating animals [28], and it might also apply to perceptions of climate change and its influence on human health. Different models of dissonance reduction strategies have been described, targeting attitudes, distraction and forgetting, denial of responsibility, and behaviour [29]. In particular, behavioural change strategies require significant effort, and they are not often experienced as comfortable or easy [26,29]. This might explain why extensive action for climate protection is often lacking, on both individual and collective levels. Addressing these barriers and strategies in decision making and public communication by policy-makers, political stakeholders and health care professionals might contribute to emphasising the urgency and need for action.

Nevertheless, in contrast to previous survey findings [16], a large proportion of participants in the present study saw and acknowledged health risks from climate change, in general. This difference may indicate the beginning of a shift in public perception regarding the association between climate change and human health. In recent years, media coverage of climate change and its consequences has increased in Germany, partly driven by the German alliance for climate change and health (KLUG e.V.) [30], and internationally through the reports of the Intergovernmental Panel on Climate Change (IPCC) [3]. The health benefits connected to climate protection, especially regarding mobility and nutrition, are topics of public interest. Thus, coverage of these benefits may contribute to increasing the public’s awareness of the association between climate change and health. In line with this, healthcare professionals are becoming increasingly visible and outspoken on this association, and this might inspire the public to improve their contributions to climate change mitigation [13,31,32,33]. Moreover, the idea that clinicians should play a more active role in responding to the climate crisis is being increasingly disseminated [34,35]. To date, it remains unclear whether—and if so how—clinicians in Germany are addressing climate change and health issues, and if they may effectively compensate for patients’ and the public’s lack of knowledge in this respect. Recent study results suggest that patients are not frequently using physicians as a source of information on climate change and health, even though they attest them high levels of trust [32]. More research is needed regarding this significant opportunity for public education and sensitisation, specifically on how physicians can be more actively involved in raising awareness and contributing to mitigation of climate change and its health impacts.

The results of the present study should be interpreted with caution, as the sample was not representative of the general population in Germany. Specifically, a large proportion of the sample was comprised of women with more advanced educational qualifications, reflecting a bias. Also, the fact that the survey was hosted by the BKK24 health insurance fund may have influenced the sample by reaching proportionately more individuals who were insured by that specific fund.

## 5. Conclusions

Concluding from the results, one solution for more action in climate protection could be appropriate risk communication, including knowledge transfer highlighting existing health risks as a result of climate change and examples of concrete options for action. This approach could incorporate the role of healthcare professionals pointing to the health benefits of sustainable behaviour, who enjoy a high level of public trust. The results also suggest that cities and councils should contribute more actively to climate protection and make their actions more visible to the population.

## Figures and Tables

**Table 1:** Demographic data of the study population (N = 697; single choices).

Variable		n	%
Sex	Female	503	72.2
	Male	190	27.3
	Diverse	4	0.6
Age group (years)	11–24	17	2.7
	25–39	113	16.2
	40–54	209	30.0
	55–64	204	29.3
	65–74	123	17.6
	>75	31	4.4
Marital status	Single	163	23.4
	Married	408	58.5
	Widowed	32	4.6
	Divorced	83	11.9
	Registered partnership	10	1.4
	Registered partner deceased	0	0.0
	Registered partnership terminated	1	0.1
Highest educational degree	No school/leaving qualification	2	0.3
	Lower secondary school	64	9.2
	Secondary school	180	25.8
	Higher secondary school	222	31.9
	University	229	32.9
Living area	Rural community (<5000 inhabitants)	198	28.4
	Small town (5000–20,000 inhabitants)	194	27.8
	Medium-sized city (20,000–100,000 inhabitants)	129	18.5
	Major city (≥100,000 inhabitants)	176	25.3

**Table 2:** Perceptions of climate change and its health consequences (N = 697).

Variable	Strongly Agree/Very Concerned (%)	Agree/Concerned (%)	Neutral (%)	Disagree/Not Concerned (%)	Strongly Disagree/Not at All Concerned (%)
**To what extent do you agree with the following statements?**					
Human-induced climate change exists * (N = 697)	62.8	22.5	9.0	2.6	3.0
Climate change has an impact on human health (N = 668)	54.2	28.6	10.8	3.6	2.8
**The following groups are affected by climate change:**					
Global population (N = 658)	80.2	14.1	2.9	1.4	1.4
European population (N = 590) *	64.6	24.1	8.5	1.7	1.2
German population (N = 590)	61.2	21.0	13.9	2.9	1.0
Myself (N = 594)	53.2	21.0	19.4	4.9	1.5
**The health of the following groups is at risk due to climate change:**					
Global population (N = 633)	66.0	22.7	8.4	2.1	0.8
European population (N = 575) *	47.1	33.6	15.8	3.1	0.3
German population (N = 576)	45.0	28.8	20.3	5.4	0.5
Myself (N = 593)	39.6	27.5	22.8	8.1	2.0
**To what extent are you concerned about:**					
Climate change (N = 666)	40.7	35.0	19.1	3.6	1.6
The health consequences of climate change (N = 632)	31.0	38.9	23.8	5.4	0.9
**To what extent are you concerned about the following risks of climate change?**					
More frequent occurrence of extreme weather events (e.g., heat waves, droughts, particulate matter) (N = 664) *	56.0	29.8	9.6	3.0	1.5
More frequent occurrence of storms and floods (N = 665) *	51.6	35.2	8.6	3.3	1.4
Higher concentration of air pollution (e.g., ozone, particulate matter) (N = 662) *	36.7	34.4	20.4	6.3	2.1
Increased contamination of water bodies with pathogens (e.g., blue-green algae) (N = 666)	33.9	33.8	21.6	8.6	2.1
Increased exposure of food to pathogens (e.g., salmonella) (N = 658)	24.8	30.4	26.3	13.5	5.0
Spread of allergenic plant species (e.g., ragweed) (N = 659) *	23.8	28.7	30.3	12.4	4.7
Spread of allergenic animal species (e.g., oak processionary moth) (N = 654)	28.3	30.7	27.4	10.1	3.5
Spread of insects carrying pathogens (e.g., dengue fever, Zika infection, West Nile fever, malaria) (N = 664)	34.3	36.4	18.2	8.4	2.6

* Differences due to rounding.

**Table 3:** Suspected or perceived risks and health consequences associated with climate change.

Variable	Yes (%)	No (%)
**Which factors do you consider relevant to climate change and its health risks?** (N = 648)		
Thermal stress due to heat	65.3	34.7
Extreme weather events (e.g., heat, storms, precipitation)	87.8	12.2
Increased occurrence of pollutants (e.g., ozone, particulate matter)	52.9	47.1
Increased UV radiation	56.0	44.0
Increased and prolonged occurrence of allergens	33.0	67.0
Propagation and spread of pathogen-carrying animals	51.9	48.1
Problems with the supply and quality of drinking water	74.2	25.8
Food hygiene problems	28.4	71.6
Degradation of bathing water quality	24.5	75.5
Rising sea levels	66.8	33.2
Social conflict	60.6	39.4
**Which health risks do you perceive as connected to climate change?** (N = 648)		
Accidents and death from extreme weather events (e.g., heat, cold, storms, landslides)	61.6	38.4
Skin cancer	51.7	48.3
Respiratory diseases	51.2	48.8
Allergies	39.6	42.6
Infectious diseases	56.2	43.8
Psychological trauma	38.6	61.4
Other	9.0	91.0
**Have you already experienced health changes connected to climate change?** (N = 627)		
Yes	114	18.2
No	340	54.2
Perhaps	173	27.6
**If yes, what are those health changes?** (N = 114)		
Accidents and their consequences	3	2.6
Infectious diseases	20	17.5
Cancer	10	8.8
Malnutrition	3	2.6
Psychological trauma	19	16.7
Allergies (e.g., new entrants, increases or extensions)	63	55.3
Respiratory diseases	41	36.0
Other (e.g., allergic reactions, circulation problems)	40	35.1

**Table 4:** Individual options for action against climate change.

Category (Examples)	Can Personally Contribute (N)	Does Personally Contribute (N)
Mobility (less car driving, more bicycling, more electric mobility, greater use of public transportation)	291	252
Resource management (thrifty/cautious/sensible use of energy, water and other resources)	195	139
Food procurement (preference for regional and seasonal food, preference for food with an organic certification, avoidance of food waste, self-supply)	179	124
Nutrition (less consumption of meat and animal products, adoption of a vegetarian and vegan diet, less consumption of processed food)	160	110
Handling of waste (package-free shopping, waste separation, recycling, consciousness of microplastics)	153	99
Consumption (upcycling, shopping second-hand, buying fewer items/clothes, minimalism, prolonged use of electronics)	134	76
Travel (no/less air travel, no/fewer cruises, vacations by train in Europe/regionally)	97	67
Use of renewable energy sources (modernisation/renovation of residential properties, use of photovoltaic plants, use of green electricity)	61	89
Biodiversity (insect-friendly planting, renaturation, tree planting, no/less use of pesticides)	50	42
Involvement and donations (participation in politics/projects, voting, support for non-governmental organisations)	24	17
Sensitisation and education (talking about climate change locally, acting as a good example, talking to kids about climate change)	22	10
Other (no fireworks, overall emissions savings, adaptation of lifestyle habits)	36	13

**Table 5:** Collective options for action against climate change.

Category (Examples)	Can City/Council Contribute (n)	Does City/Council Contribute (n)
Traffic and mobility (more/better/safer bikeways, expansion of public transportation, car-free cities, more charging stations, car sharing, speed limits, greater traffic control)	242	63
Modernisation and extension of renewable energy sources (photovoltaic plants on public buildings, benefits for private reconstruction, standards for building extensions)	141	32
Green spaces and planting (more tree planting, less logging, more parks, more community gardens, more conservation areas, renaturation, green roofing)	139	25
Sustainable construction (less impervious surfaces, eco-friendly building regulations, fewer parking spaces, regulations for rockeries)	71	11
Resource saving (public lighting, public fountains and sprinkler installations, incentives to save resources [e.g., CO_2_ taxes], bans and bids)	62	12
Education, sensitisation, consultation and inclusion (information in schools and kindergartens, public campaigns, more frequent referendums)	47	7
Food procurement and the promotion of regional offers (regulations for food prices [e.g., price increases for meat products and price decreases for regional vegetables], regulations to prohibit waste)	29	0
Support for projects and initiatives (demonstrations, days of action, litter clean-up events, funding for eco-friendly companies)	27	16
Waste and packaging (prohibitions/taxes on plastics, more public waste bins, incentives for waste avoidance, recycling)	23	5
Adjustment of climate change and anticipatory action (preparations for heat waves, more shaded places in cities, flood prevention, secure water supplies)	21	3
Adaptation of cultural offerings and living spaces (river bathing, playgrounds, drinking water fountains)	19	2
Agriculture (reserve areas, promotion of sustainable processes and organic cultivation, improvement of animal welfare)	11	1
Adaptation of consumption (promotion of second-hand stores, incentives and legal framework for less consumption, adaptation of food in canteens)	8	2
Other (sanctions for non–eco-friendly industries and companies, improvement of air quality, hiring of personnel responsible for climate protection in administration)	23	4

## Data Availability

The datasets generated and/or analysed during the current study are available from the corresponding author upon reasonable request.

## References

[1] Watts N., Amann M., Arnell N., Ayeb-Karlsson S., Beagley J., Belesova K., Boykoff M., Byass P., Cai W., Campbell-Lendrum D. (2021). The 2020 report of The Lancet Countdown on health and climate change: Responding to converging crises. Lancet.

[2] Watts N., Amann M., Arnell N., Ayeb-Karlsson S., Belesova K., Boykoff M., Byass P., Cai W., Campbell-Lendrum D., Capstick S. (2019). The 2019 report of The Lancet Countdown on health and climate change: Ensuring that the health of a child born today is not defined by a changing climate. Lancet.

[3] Intergovernmental Panel on Climate Change (IPCC) (2022). Climate Change 2022: Impacts, Adaption and Vulnerability.

[4] Steffen W., Richardson K., Rockström J., Cornell S.E., Fetzer I., Bennett E.M., Sörlin S. (2015). Planetary boundaries: Guiding human development on a changing planet. Science.

[5] Ripple W.J., Wolf C., Gregg J.W., Levin K., Rockström J., Newsome T.M., Lenton T.M. (2022). World Scientists’ Warning of a Climate Emergency 2022. BioScience.

[6] Romanello M., Di Napoli C., Drummond P., Green C., Kennard H., Lampard P., Scamman D., Arnell N., Ayeb-Karlsson S., Ford L.B. (2022). The 2022 report of the Lancet Countdown on health and climate change: Health at the mercy of fossil fuels. Lancet.

[7] Maibach E.W., Kreslake J.M., Roser-Renouf C., Rosenthal S., Feinberg G., Leiserowitz A.A. (2015). Do Americans Understand That Global Warming Is Harmful to Human Health? Evidence From a National Survey. Ann. Glob. Health.

[8] Parise I. (2018). A brief review of global climate change and the public health consequences. Aust. J. Gen. Pract..

[9] Parker C.L., Wellbery C.E., Mueller M. (2019). The Changing Climate: Managing Health Impacts. Am. Fam. Physician.

[10] World Health Organization Climate Change and Health. https://www.who.int/news-room/fact-sheets/detail/climate-change-and-health.

[11] Karliner J., Slotterback S., Arup Boyd R., Ashby B., Steele K. Health Care Without Harm. Health Care’s Climate Footprint—How the health sector contributes to the global climate crisis and opportunities for action. https://noharm-global.org/sites/default/files/documents-files/5961/HealthCaresClimateFootprint_092319.pdf.

[12] Storz M.A. (2018). A Practical Guide for Physicians and Health Care Workers to Reduce Their Carbon Footprint in Daily Clinical Work. Perm. J..

[13] Mezger N.C.S., Thöne M., Wellstein I., Schneider F., Litke N., Führer A.G., Clar C., Kantelhardt E.J. (2021). Climate protection in practices—Current status, motivation and challenges in outpatient care. Z. Evid. Fortbild. Qual. Gesundhwes.

[14] Minogue V., Wells B. (2016). Managing resources and reducing waste in healthcare settings. Nurs. Stand..

[15] Nicolet J., Mueller Y., Paruta P., Boucher J., Senn N. (2022). What is the carbon footprint of primary care practices? A retrospective life-cycle analysis in Switzerland. Environ. Health.

[16] Berger N., Lindemann A.K., Böl G.F. (2019). Public perception of climate change and implications for risk communication. Bundesgesundheitsblatt.

[17] Akerlof K., Debono R., Berry P., Leiserowitz A., Roser-Renouf C., Clarke K.-L., Rogaeva A., Nisbet M.C., Weathers M.R., Maibach E.W. (2010). Public perceptions of climate change as a human health risk: Surveys of the United States, Canada and Malta. Int. J. Environ. Res. Public Health.

[18] de Freitas C. (2017). Public and patient participation in health policy, care and research. Porto. Biomed. J..

[19] Hsieh H.F., Shannon S.E. (2005). Three approaches to qualitative content analysis. Qual. Health Res..

[20] Amuasi J.H., Lucas T., Horton R., Winkler A.S. (2020). Reconnecting for our future: The Lancet One Health Commission. Lancet.

[21] Moser S.C. (2010). Communicating climate change: History, challenges, process and future directions. WIREs Climate Chang..

[22] Moser S.C. (2016). Reflections on climate change communication research and practice in the second decade of the 21st century: What more is there to say?. WIREs Climate Chang..

[23] Michaels D., Monforton C. (2005). Manufacturing uncertainty: Contested science and the protection of the public’s health and environment. Am. J. Public Health.

[24] Mccright A.M., Dunlap R.E. (2014). Challenging Global Warming as a Social Problem: An Analysis of the Conservative Movement’s Counter-claims. Soc. Probl..

[25] Visschers V.H.M. (2018). Public Perception of Uncertainties Within Climate Change Science. Risk Anal..

[26] Festinger L. (1957). A Theory of Cognitive Dissonance.

[27] Klein J., McColl G. (2019). Cognitive dissonance: How self-protective distortions can undermine clinical judgement. Med. Educ..

[28] Loughnan S., Bastian B., Haslam N. (2014). The Psychology of Eating Animals. Curr. Dir. Psychol. Sci..

[29] McGrath A. (2017). Dealing with dissonance: A review of cognitive dissonance reduction. Soc. Personal. Psychol. Compass.

[30] Deutsche Allianz Klimawandel und Gesundheit (KLUG) Gemeinsam Handeln Für KLIMA und Gesundheit: Aufklärung Und Agendasetting. https://www.klimawandel-gesundheit.de/aufklarung-und-agendasetting/.

[31] André H., Holguera J.G., Depoux A., Pasquier J., Haller D.M., Rodondi P.-Y., Schwarz J., Senn N. (2022). Talking about Climate Change and Environmental Degradation with Patients in Primary Care: A Cross-Sectional Survey on Knowledge, Potential Domains of Action and Points of View of General Practitioners. Int. J. Environ. Res..

[32] Boland T.M., Temte J.L. (2019). Family Medicine Patient and Physician Attitudes Toward Climate Change and Health in Wisconsin. Wilderness Environ. Med..

[33] Moniz M.A., Daher D.V., Sabóia V.M., Ribeiro C.R.B. (2020). Environmental health: Emancipatory care challenges and possibilities by the nurse. Rev. Bras. Enferm..

[34] Crowley R., Daniel H., Cooney T.G., Engel L.S., Health, Public Policy Committee of the American College of Physicians (2020). Envisioning a Better U.S. Health Care System for All: Coverage and Cost of Care. Ann. Intern. Med..

[35] Veidis E.M., Myers S.S., Almada A.A., Golden C.D. (2019). A call for clinicians to act on planetary health. Lancet.

